# A FAERS pharmacovigilance analysis of dementia-related adverse event reports

**DOI:** 10.1097/MD.0000000000051134

**Published:** 2026-10-16

**Authors:** Tiantian Tao, Bingyao Liu, Chao Tao, Weiyi Chen, Ruixiao Li, Shiying Mao, Ke Wang, Meiling Zhou, Xianbin Liu

**Affiliations:** a Department of Gastroenterology, Mianyang Central Hospital, School of Medicine, University of Electronic Science and Technology of China, Mianyang, Sichuan, China; b Department of Radiology, West China Hospital Sichuan University Jintang Hospital, Jintang First People’s Hospital, Chengdu, China; c Department of Pharmacy, The Affiliated Hospital, Southwest Medical University, Luzhou, China; d Department of Clinical Pharmacy, School of Pharmacy, Southwest Medical University, Luzhou, China; e College of Pharmaceutical Sciences, Guizhou University of Traditional Chinese Medicine, Guiyang, China; f Department of Pharmacy, Mianyang Central Hospital, School of Medicine, University of Electronic Science and Technology of China, Mianyang, Sichuan, China.

**Keywords:** disproportionate analysis, drug-related dementia, FAERS, immunosuppressants, pharmacovigilance, psycholeptics

## Abstract

Dementia-related adverse event (AE) reports represent an important area of concern in pharmacovigilance, particularly considering the clinical relevance of cognitive impairment and the complexity of medication use among older adults. This study aims to systematically characterize the reporting patterns of dementia-related AEs and detect disproportionality signals. Dementia-related AE reports in the Food and Drug Administration Adverse Event Reporting System database from the first quarter of 2004 to the first quarter of 2024 were analyzed using the reporting odds ratio and proportional reporting ratio methods. The reports were classified and organized at the Standardized Medical Dictionary for Regulatory Activities Queries and Preferred Term levels to explore the potential association strength between drug-associated dementia and the related drugs and drug categories. We identified a total of 33,772 AE reports related to dementia, with 1103 drugs reported as the main suspected drugs. Overall, immunosuppressants, psycholeptics, and antineoplastic agents were the most frequently reported drug categories. Among them, apixaban exhibited the highest number of AE reports, followed by pimavanserin and lenalidomide. Notably, psycholeptics represented the highest proportion of the top 30 drugs by AE report count. Disproportionality analysis showed that 191 and 135 drugs were associated with potential signals of dementia. This study provides a comprehensive overview of dementia-related AE reports from a pharmacovigilance perspective, offering pharmacovigilance signals that may inform further drug safety evaluation.

## 1. Introduction

As human life expectancy continues to increase and the global population ages, the detection and monitoring of adverse events (AEs) caused by medication have become increasingly important. In this context, drug-related dementia, a serious AE, has garnered widespread attention. This condition not only imposes significant psychological and economic burdens on patients and their families but also constitutes a major global public health issue. Therefore, heightened vigilance and research on dementia events associated with drug exposure are urgently needed.

In an era of rapid introduction of new drugs, although rigorous safety assessments have been conducted during clinical trials, certain rare or long-term AEs may not be captured promptly. Consequently, utilizing real-world data for pharmacovigilance is crucial to ensuring patient safety and improving clinical outcomes. The Food and Drug Administration Adverse Event Reporting System (FAERS) database, the largest spontaneous AE reporting database globally, plays a critical role in collecting and analyzing AEs associated with marketed drugs and therapeutic biologics. With over 28 million AE reports accumulated and more than 1 million new reports added annually,^[1,2]^ FAERS serves as an essential tool for evaluating the relationships between drugs and drug-related AEs based on real-world data. By employing data mining algorithms, researchers can quantitatively detect signals from the FAERS database, thereby facilitating pharmacovigilance analysis.^[3]^

Dementia is a chronic, progressive, and incurable clinical syndrome resulting from various direct and indirect factors that damage the brain, leading to a comprehensive decline in cognitive functions. It typically manifests as progressive and diffuse brain dysfunction, particularly deteriorating memory, learning, and abstract thinking.^[4,5]^ Research indicates that the prevalence of dementia among individuals aged 65 and over in China is as high as 6.9%,^[6]^ with over 57 million people currently affected worldwide.^[7]^ Due to the increasing number of elderly individuals, nearly 10 million new cases are reported each year, and it is projected to increase 3-fold by 2050.^[8,9]^ Dementia imposes a significant economic burden on society. In 2010, the global economic loss due to dementia was $604 billion, which increased to $818 billion by 2015.^[10]^ According to World Health Organization statistics, the economic loss due to dementia reached $1.3 trillion in 2019,^[11]^ marking a 115.23% increase over 9 years.

This study aims to utilize the FAERS database to conduct real-world pharmacovigilance research on drug-related dementia. We screened data from the FAERS database covering the period from the first quarter of 2004 to the first quarter of 2024, identifying relevant AE reports and compiling a list of drugs linked to an increased risk of dementia. In addition, we performed signal detection to explore potential associations between medication exposure and dementia-related AEs, thereby uncovering possible drug risks. Our objective is to provide clinical evidence and drug safety information for the prevention and treatment of drug-associated dementia. Our findings are intended to serve as a reference for clinical practice and drug regulation, promoting the rational use of medications and ensuring patient safety.

## 2. Materials and methods

### 2.1. Data source

This study leverages the FAERS database to conduct pharmacovigilance research on dementia as an AE. The FAERS database, a spontaneous reporting system and open data platform, compiles safety reports on drugs and therapeutic biologics submitted by patients, healthcare professionals, and pharmaceutical companies. Since 2004, the FAERS database has publicly released all AE reports received by the Food and Drug Administration (FDA), updated quarterly. It includes 7 datasets: patient demographic and administrative information (DEMO), adverse event information (REAC), drug information (DRUG), drug therapy start and end dates (THER), patient outcome information (OUTC), report source information (RPSR), and indications for use/diagnosis (INDI).

For this study, we extracted data from the FAERS database covering the first quarter of 2004 to the first quarter of 2024. The data were screened and processed according to FDA guidelines using SAS 9.4 software (Fig. S1A, Supplemental Digital Content 1). Duplicate reports were processed according to the FDA-recommended method. For reports sharing the same CASEID, only the record with the most recent FDA receipt date (FDA_DT) was retained. When FDA_DT was identical, the record with the largest PRIMARYID was retained. Drug names were standardized before analysis, and variations arising from differences in capitalization, spacing, abbreviations, brand or generic names, and dosage forms were harmonized and mapped to the corresponding active ingredient names. This study included only reports in which the drug of interest was designated as the primary suspect drug. Reports classified as secondary suspect, concomitant, or interacting were excluded from the primary signal analysis. Regarding missing data, reports lacking key identification information or for which deduplication or linkage across data tables could not be completed were excluded. Missing values for variables such as age, sex, reporting country, reporter type, and clinical outcome were not imputed but were recorded as “unknown” or “missing.”

### 2.2. Identifying relevant reports

In this study, AEs were coded using preferred terms (PTs) from the Medical Dictionary for Regulatory Activities (MedDRA) version 26.1. Each PT can correspond to multiple high-level terms, high-level group terms, and system organ classes. In addition, standardized MedDRA queries (SMQs) are integrated tools within MedDRA that comprise a collection of PTs that indicate similar medical conditions, thereby facilitating the identification and retrieval of relevant safety reports.

To ensure specificity and accuracy in identifying dementia-related AE reports, MedDRA version 26.1 was used as the standardized terminology throughout the study period from the first quarter of 2004 to the first quarter of 2024. PTs included in the narrow-scope search of the “Dementia (SMQ)” were selected based on MedDRA version 26.1 to identify dementia-related AE reports. The specific PTs used were listed in Table 1.

**Table 1:** PTs included in the narrow-scope search of “Dementia (SMQs).”

MedDRA code	PTs
10070997	Korsakoff syndrome
10012271	Dementia Alzheimer’s type
10064199	Variant Creutzfeldt–Jakob disease
10012267	Dementia
10012293	Dementia of the Alzheimer’s type, uncomplicated
10068968	Frontotemporal dementia
10081973	Hippocampal atrophy
10063629	Hippocampal sclerosis
10075174	Mixed dementia
10065424	Mini-mental status examination abnormal
10036813	Progressive supranuclear palsy
10011384	Creutzfeldt–Jakob disease
10039966	Senile dementia
10079991	Clinical dementia rating scale score abnormal
10067889	Dementia with Lewy bodies
10078208	Corticobasal degeneration
10079301	Prion disease
10077237	Scatolia
10012295	Dementia of the Alzheimer’s type, with delusions
10057678	Vascular dementia
10012296	Dementia of the Alzheimer’s type, with depressed mood
10078036	Early onset familial Alzheimer’s disease
10036631	Presenile dementia
10012294	Dementia of the Alzheimer’s type, with delirium

MedDRA = medical dictionary for regulatory activities, PTs = preferred terms, SMQ = standardized MedDRA query.

### 2.3. Analytical methods

In this study, we employed a descriptive analysis to summarize the clinical characteristics of patients with drug-related dementia, including the report year, patient age, gender, reporter identity, reporting country, and outcomes. Disproportionality analysis, a data mining algorithm, was used to quantitatively detect AE signals in large pharmacovigilance databases. It should be emphasized that disproportionality analysis can only identify disproportionate reporting signals, indicating a potential statistical association in reporting patterns between a drug and an AE. However, it cannot establish a causal relationship. Utilizing the classic 2 × 2 contingency table (Table S1, Supplemental Digital Content 2), this analysis compares the frequency of the target drug-AE pair with the background frequency to establish statistical associations.

Specifically, we employed the reporting odds ratio (ROR) and proportional reporting ratio (PRR) methods to detect disproportionality signals of dementia-related AEs. The calculation formulas and evaluation criteria are shown in Table 2. Higher ROR or PRR values indicate greater disproportionality in the reporting of dementia-related AEs for a given drug and may therefore help identify potential safety signals warranting further investigation. However, because FAERS lacks reliable denominator data on drug exposure, these measures cannot be directly interpreted as estimates of, or used to compare, the true clinical risks across different drugs.

**Table 2:** Overview of algorithms utilized for signal detection.

Algorithms	Formulas	Criteria
ROR	$ROR\;\;\;=\;\;\;\frac{ad}{bc}$	Lower limit of 95% CI > 1, *a* ≥ 3
	$95\;\%\;\;\;CI\;\;\;=\;\;\;e^{ln\left(ROR\right)\;\;\;\pm \;\;\;1\mathrm{.96}\;\;\;{\left(\frac{1}{a}\;\;\;+\;\;\;\frac{1}{\;\;\;b}\;\;\;+\;\;\;\frac{1}{c}\;\;\;+\frac{1}{d}\right)}^{0\mathrm{.5}}}$	
PRR	$PRR\;\;\;=\;\;\;\frac{a/(a+b)}{c/(c+d)}$	PRR ≥ 2, χ^2^ ≥ 4, *a* ≥ 3
	$\chi^{2}\;\;\;=\;\;\;\frac{{\left(ad-bc\right)}^{2}(a+b+c+d)}{(a+b)(c+d)(a+c)(b+d)}$	

CI = confidence interval, PRR = proportional reporting ratio, ROR = reporting odds ratio, χ^2^ = chi-squared.

## 3. Results

### 3.1. Descriptive analysis

From the first quarter of 2004 to the first quarter of 2024, the FAERS database included 33,002 patients with reports related to dementia. Of these, 33,772 reports were identified as target AE reports through a narrow-scope search using the “Dementia (SMQs).” The number of dementia-related AE reports exhibited a general upward trend over the years, peaking in 2021 with a total of 3209 cases (Fig. 1A). Gender analysis revealed that 53.00% of the reports were from female patients, higher than the 40.79% observed in male patients (Fig. 1B). In terms of age, the highest incidence was among those aged over 65, accounting for 46.72% of the total (Fig. 1C). Regarding the geographic distribution, the majority of dementia reports originated from the United States (57.18%; Fig. 1D). The primary sources of these submissions were consumers (56.20%), followed by physicians (19.71%) and pharmacists (11.26%; Fig. 1E). In addition, the most common treatment outcome was initial or prolonged hospitalization, accounting for 28.29% of all outcomes, followed by death (14.79%), disability (4.76%), and life-threatening events (2.37%; Fig. 1F).

**Figure 1.**
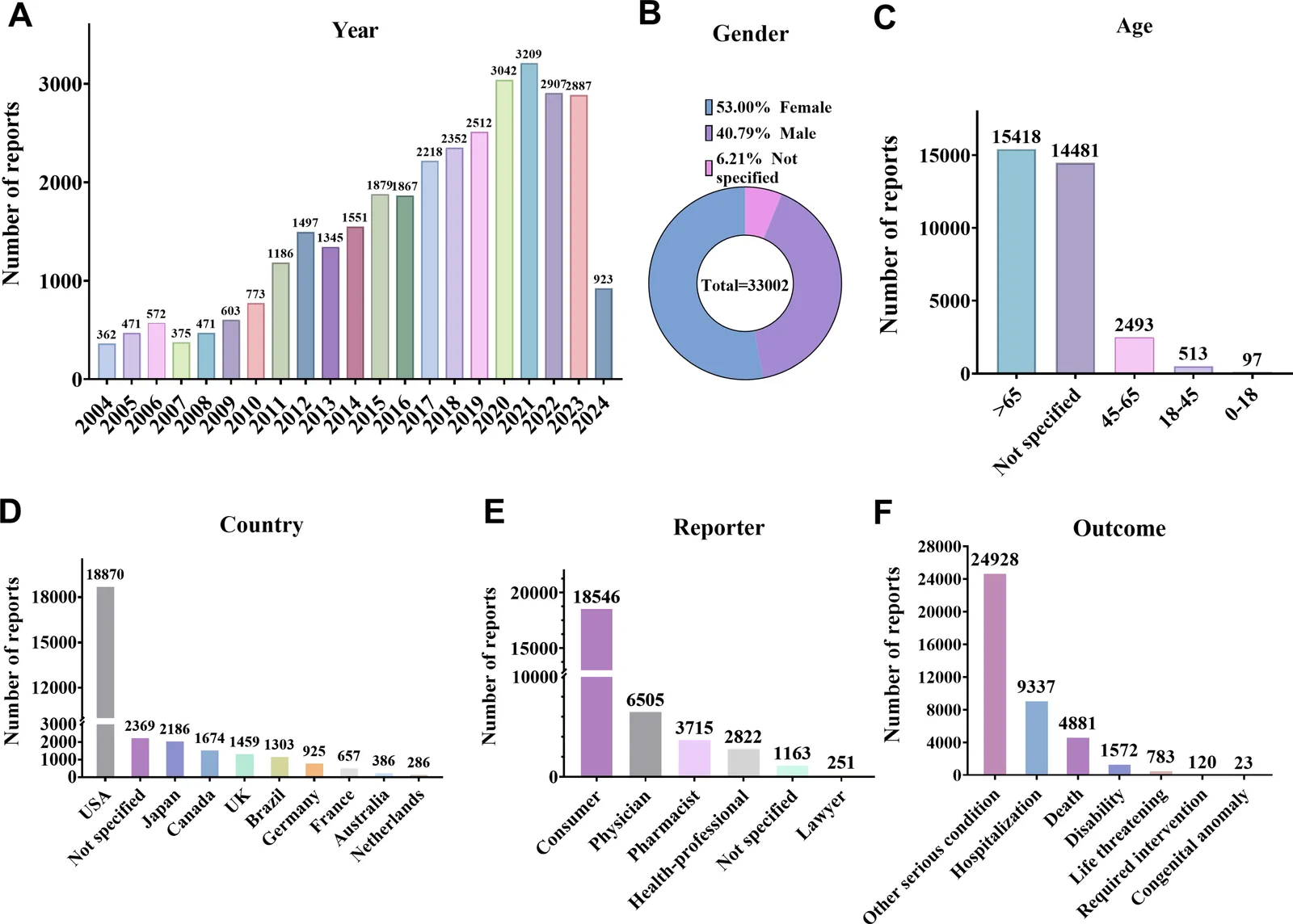
Overview of AE reports related to dementia in the FAERS database. (A) Annual number of AE reports. (B) Gender distribution of patients. (C) Age distribution of patients. (D) Top 10 countries by number of reports. (E) Occupational distribution of reporters. (F) Outcome distribution of AEs in patients. AE = adverse event, FAERS = Food and Drug Administration Adverse Event Reporting System.

### 3.2. Identification of drugs associated with dementia

To summarize the distribution of primary suspect drugs and detect disproportionality signals, we screened the 33,772 dementia-related AE reports. After excluding non-primary suspect drugs, those lacking generic names, duplicate records, and drugs with unclear names, we finalized a list of 1103 drugs. These drugs were reported as primary suspect drugs in association with the target AE in at least one report. Using the narrow-scope search for “Dementia (SMQ),” the PT with the highest number of AE reports was dementia (22,508/33,772), followed by dementia Alzheimer’s type (8042/33,772), vascular dementia (752/33,772), dementia with Lewy bodies (714/33,772), senile dementia (587/33,772), frontotemporal dementia (360/33,772), Creutzfeldt–Jakob disease (263/33,772), and progressive supranuclear palsy (169/33,772; Fig. 2A). The number of drugs associated with these 8 PTs was 992, 622, 227, 202, 187, 156, 126, and 78, respectively (Fig. 2B). In addition, the number of causative drugs for PTs such as scatolia, variant Creutzfeldt–Jakob disease, prion disease, early onset familial Alzheimer’s disease, and clinical dementia rating scale score abnormal were all fewer than ten.

**Figure 2.**
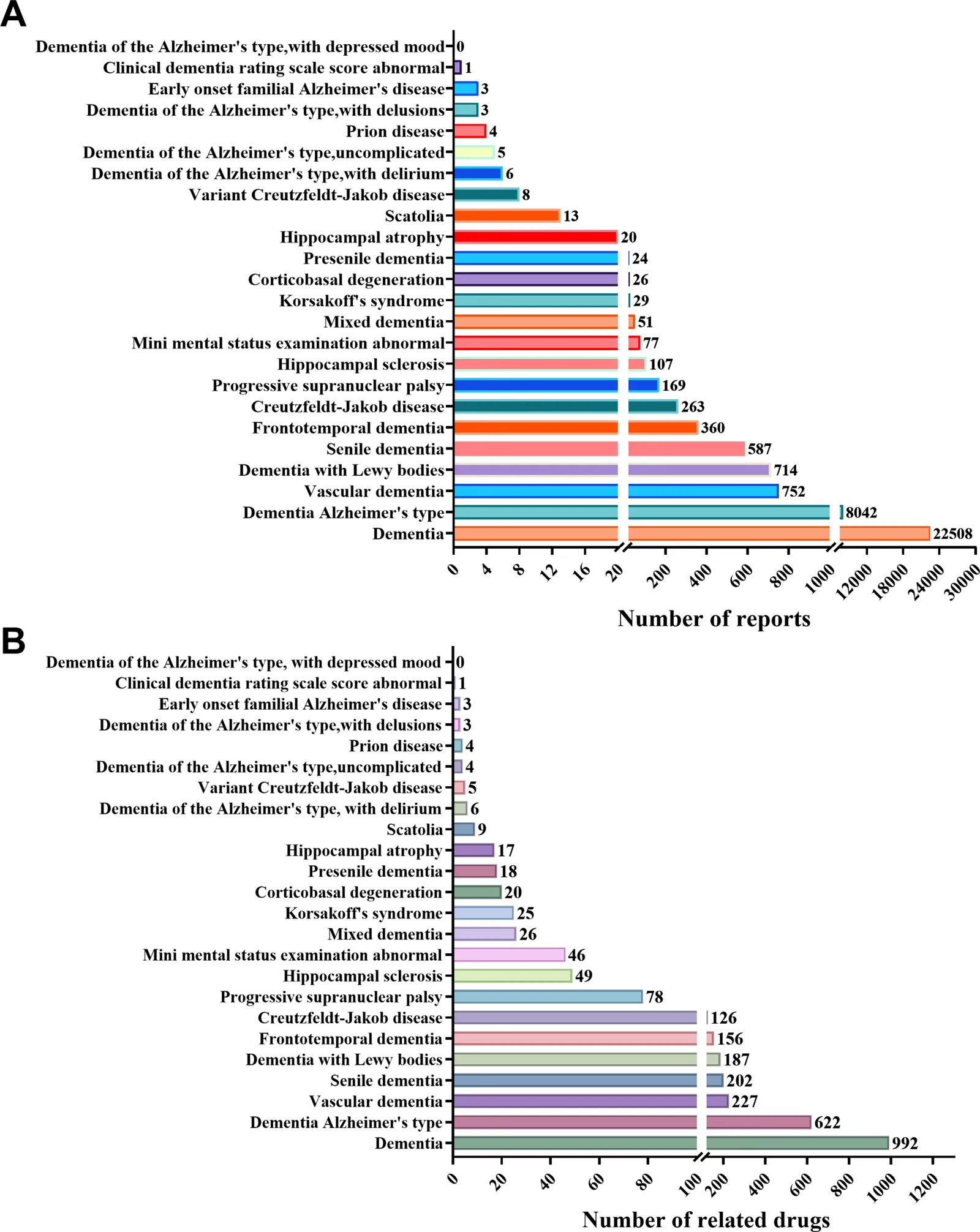
Number of reports (A) and number of related drugs (B) on PTs. PTs = preferred terms.

### 3.3. Proportional distribution of drug classes in AE reports

In this study, we utilized the Anatomical Therapeutic Chemical classification system to categorize 1103 potential drugs into pharmacological or therapeutic subgroups (second level of Anatomical Therapeutic Chemical), covering 77 drug classes. Among these, the top 10 drug classes with the highest number of related drugs are immunosuppressants (n = 4734), psycholeptics (n = 3313), antineoplastic agents (n = 2626), psychoanaleptics (n = 2480), antithrombotic agents (n = 2423), drugs used in diabetes (n = 2288), antiepileptics (n = 1476), agents acting on the renin-angiotensin system (n = 1277), antiparkinson drugs (n = 1255), and drugs for treatment of bone diseases (n = 1161). We further mapped the 1103 medications to specific PTs, revealing notable differences in the drug categories involved across different PTs. PTs with more than 100 reports are presented in Figure 3. At the PT level, antiepileptics accounted for the largest proportion of reports related to “hippocampal sclerosis” (n = 47, 43.93%). Psycholeptics represented the predominant drug category among reports of “dementia with Lewy bodies” (n = 315, 44.12%). In addition, the dementia (PT) subgroup included the highest number of drug categories (75 classes). In contrast, the subgroups related to prion disease (PT), early onset familial Alzheimer’s disease (PT), and clinical dementia rating scale score abnormal (PT) each have fewer than 5 classes of associated pathogenic drugs.

**Figure 3.**
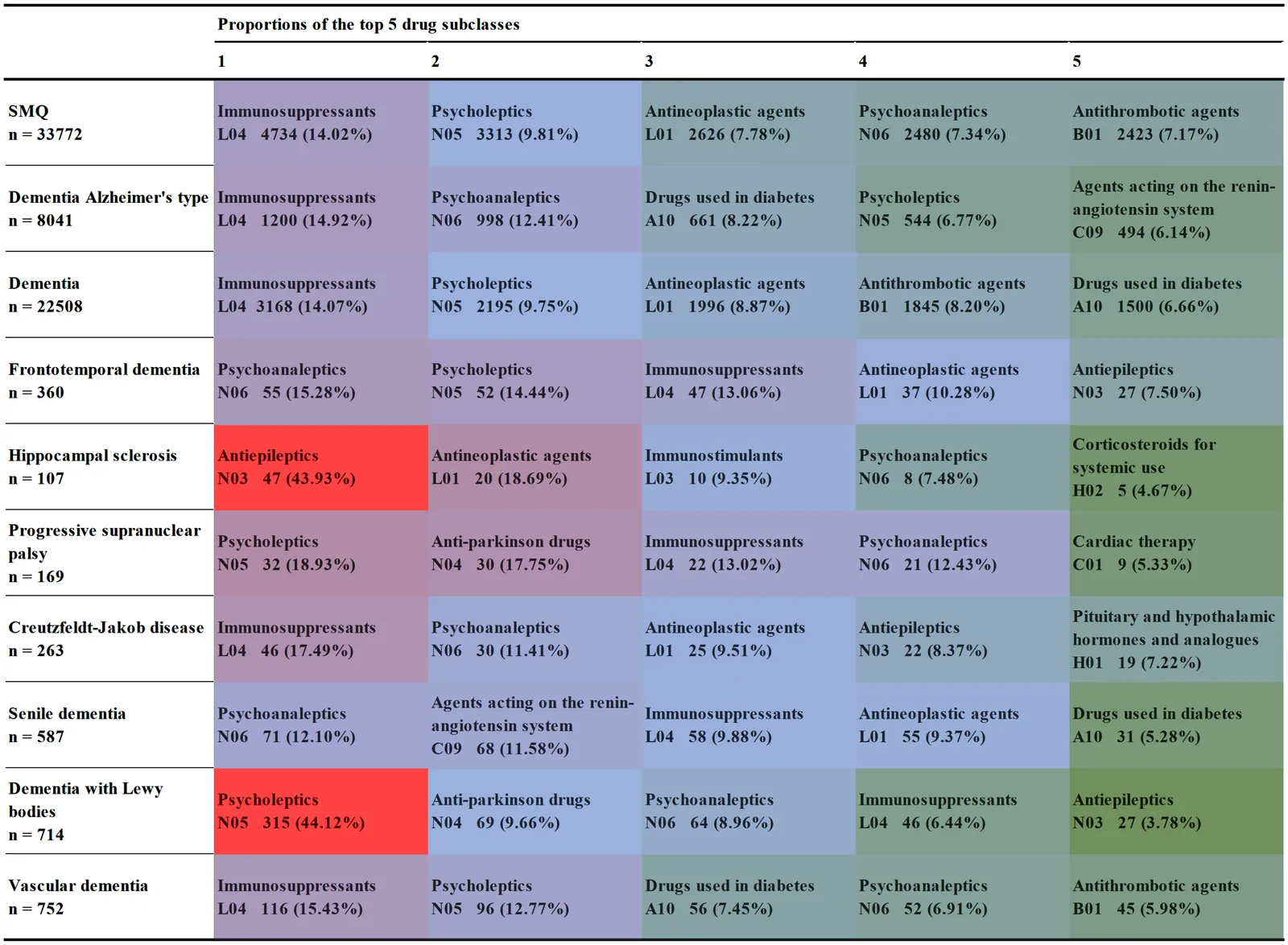
Distribution of the top 5 drug categories at the SMQ and PT levels (PTs with ≤100 reports omitted). PT = preferred term, SMQ = standardized MedDRA query.

### 3.4. Proportional distribution of drugs in AE reports

We analyzed the distribution of AE reports for 1103 drugs, focusing on the top 30 most frequently reported drugs at both the SMQ and PT levels, as depicted in Figure 4. At the SMQ level, apixaban accounted for the highest proportion of reports (4.93%), followed by pimavanserin (2.81%), lenalidomide (2.53%), adalimumab (2.27%), carbidopa/levodopa (2.22%), insulin glargine (1.89%), quetiapine (1.73%), sacubitril/valsartan (1.66%), etanercept (1.64%), and insulin lispro (1.54%). These are the top 10 drugs with the highest proportions of AE reports. Overall, among the top 30 drugs, psycholeptics were the most frequently reported drug category (6/30), followed by immunosuppressive agents (ISAs, 4/30). Other prevalent categories included antithrombotic agents (3/30) and drugs for the treatment of bone diseases (3/30). It should be emphasized that the number of AE reports is influenced by multiple factors, including drug utilization patterns, disease prevalence, clinical or media attention, and variations in reporting practices. Therefore, a higher number of reports does not necessarily indicate a higher underlying clinical risk.

**Figure 4.**
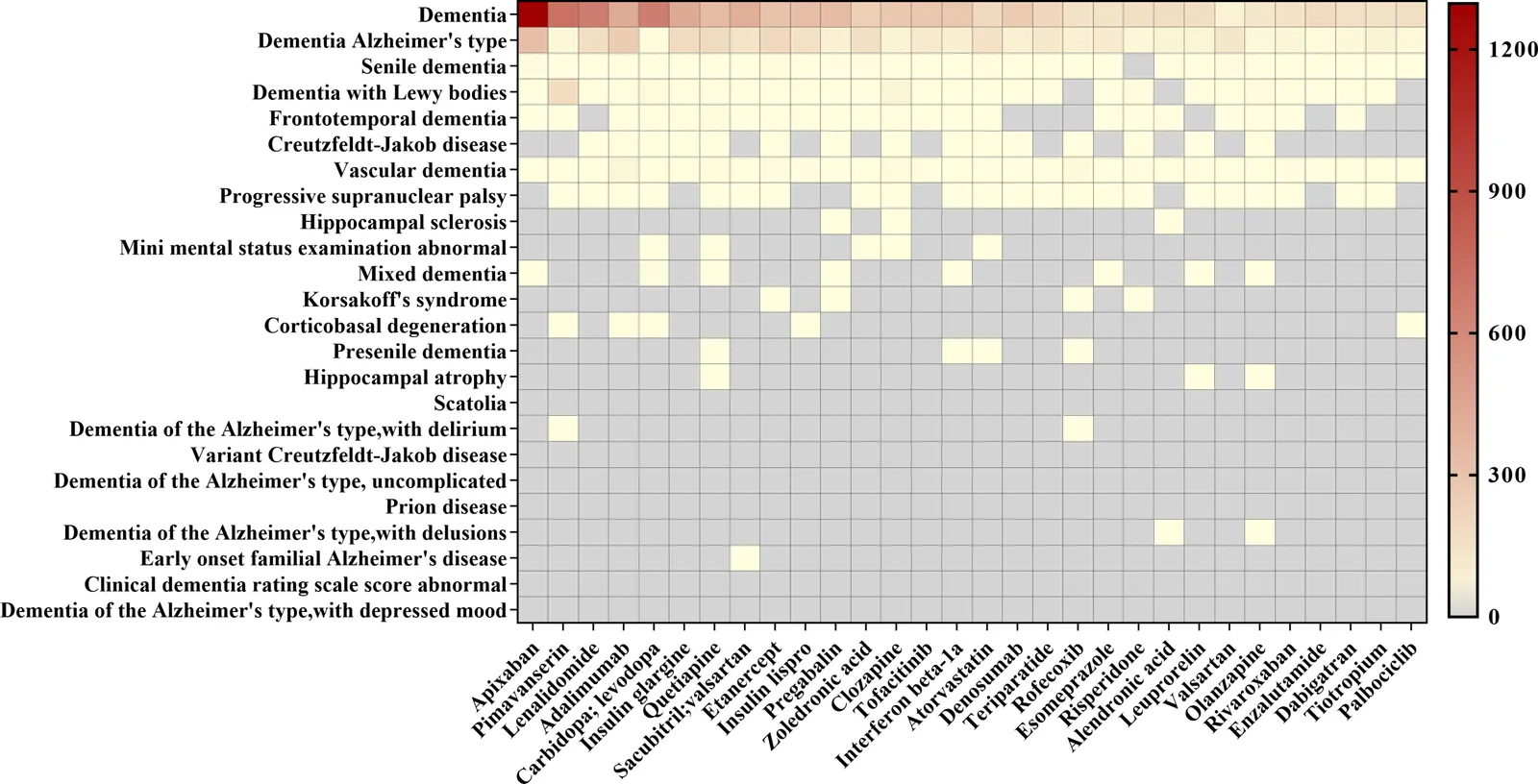
Reporting proportion of the top 30 highest agents at the SMQ and PT levels. Red blocks indicate significant associations between dementia and SMQs, while gray blocks indicate nonsignificant associations. PT = preferred term, SMQ = standardized MedDRA query.

### 3.5. AE signal detection results

To assess the potential risk of dementia for the 1103 drugs, we conducted comprehensive AE signal detection, with the results presented in Table S2, Supplemental Digital Content 3. To minimize potential bias caused by confounding by indication, 8 antidementia medications, such as rivastigmine and donepezil, were excluded from the primary analysis. Table 3 highlights the AE signal detection results for the top 30 drugs. The 3 drugs with the highest number of reports include apixaban (N = 1664 cases, ROR = 11.26, 95% confidence interval [CI] = 10.71–11.83, PRR = 11.19, χ^2^ = 14,682.21), pimavanserin (N = 948 cases, ROR = 15.87, 95% CI = 14.87–16.93, PRR = 15.72, χ^2^ = 12,711.63), and lenalidomide (N = 853 cases, ROR = 1.94, 95% CI = 1.81–2.07, PRR = 1.94, χ^2^ = 376.15). Conversely, adalimumab, despite being ranked fourth in the number of reports (N = 766 cases, ROR = 0.62, 95% CI = 0.58–0.67, PRR = 0.62, χ^2^ = 172.91), demonstrated a negative signal. Considering both the results of ROR and PRR, 191 and 135 drugs, respectively, indicated positive signals. All 135 drugs with positive PRR signals are also included in the list of drugs with positive signals in ROR (Fig. S1B, Supplemental Digital Content 1). In addition, we reviewed the drug leaflets for the top 30 medications and found that only 6 of them listed dementia as an AE or mentioned it in their precautions. Notably, 5 of these drugs belong to the category of psycholeptics.

**Table 3:** AE signal detection results of the top 30 drugs associated with dementia, based on ROR and PRR.

Drug name	No. of reports	ROR (95% CI)	Signal	PRR (χ^2^)	Signal	Drug leaflet
Apixaban	1664	11.26 (10.71–11.83)	Y	11.19 (14,682.21)	Y	N
Pimavanserin	948	15.87 (14.87–16.93)	Y	15.72 (12,711.63)	Y	Y
Lenalidomide	853	1.94 (1.81–2.07)	Y	1.94 (376.15)	N	N
Adalimumab	766	0.62 (0.58–0.67)	N	0.62 (172.91)	N	N
Carbidopa/Levodopa	750	5.79 (5.38–6.22)	Y	5.77 (2892.07)	Y	Y
Insulin glargine	637	4.45 (4.11–4.81)	Y	4.44 (1665.98)	Y	N
Quetiapine	584	3.17 (2.92–3.44)	Y	3.17 (852.19)	Y	Y
Sacubitril/Valsartan	562	3.21 (2.96–3.49)	Y	3.21 (840.64)	Y	N
Etanercept	555	0.62 (0.57–0.68)	N	0.62 (125.12)	N	N
Insulin lispro	521	4.11 (3.77–4.48)	Y	4.1 (1204.59)	Y	N
Pregabalin	448	1.89 (1.72–2.07)	Y	1.89 (184.52)	N	N
Zoledronic acid	436	2.54 (2.31–2.79)	Y	2.54 (401.98)	Y	N
Clozapine	435	2.73 (2.48–3.00)	Y	2.72 (468.57)	Y	Y
Tofacitinib	410	1.72 (1.56–1.89)	Y	1.72 (121.06)	N	N
Interferon beta-1a	385	1.21 (1.10–1.34)	Y	1.21 (14.37)	N	N
Atorvastatin	375	2.49 (2.25–2.76)	Y	2.49 (330.22)	Y	N
Denosumab	357	1.77 (1.60–1.97)	Y	1.77 (118.57)	N	N
Teriparatide	340	1.44 (1.30–1.60)	Y	1.44 (45.53)	N	N
Rofecoxib	276	1.79 (1.59–2.02)	Y	1.79 (96.13)	N	N
Esomeprazole	264	1.57 (1.39–1.77)	Y	1.57 (54.09)	N	N
Risperidone	251	1.75 (1.55–1.98)	Y	1.75 (80.43)	N	Y
Alendronic acid	237	1.19 (1.05–1.35)	Y	1.19 (6.98)	N	N
Leuprorelin	222	1.88 (1.65–2.14)	Y	1.88 (90.57)	N	N
Valsartan	221	3.58 (3.14–4.09)	Y	3.57 (407.13)	Y	N
Olanzapine	219	1.85 (1.62–2.11)	Y	1.85 (84.36)	N	Y
Rivaroxaban	219	1.31 (1.14–1.49)	Y	1.31 (15.71)	N	N
Enzalutamide	217	2.58 (2.26–2.95)	Y	2.58 (208.67)	Y	N
Dabigatran	214	2.11 (1.84–2.41)	Y	2.10 (123.30)	Y	N
Tiotropium	212	2.32 (2.02–2.65)	Y	2.31 (157.27)	Y	N
Palbociclib	210	1.36 (1.19–1.56)	Y	1.36 (19.9)	Y	N

Signal: Y, positive signal; N, negative signal. Drug leaflet: Indicates whether dementia is listed as an AE or mentioned in the precautions (Y, yes; N, no). Differences between ROR and PRR results are attributable to their different signal detection criteria, which primarily arise from the additional χ^2^ requirement and PRR criteria applied in PRR-based signal detection. Therefore, some drugs may meet the criteria for a positive ROR signal but not for a positive PRR signal. This table excludes antidementia medications. A more comprehensive dataset, including all analyzed drugs, is provided in Table S2, Supplemental Digital Content 3.

AE = adverse event, CI = confidence interval, PRR = proportional reporting ratio, ROR = reporting odds ratio.

## 4. Discussion

As the global trend of population aging intensifies, drug-related dementia has become a prominent concern in drug safety, posing significant challenges to healthcare systems. This study leverages the FAERS database and employs disproportionality analysis (using ROR and PRR) to quantify the strength of the reporting association between various drugs and dementia-related AEs. We summarized 33,772 reports of dementia-related AEs associated with drug exposure, identifying 1103 drugs associated with dementia. This research also outlined the fundamental clinical characteristics of dementia-related AE reports. Building on this foundation, we investigated the proportion of reports for different drugs and drug categories, conducted adverse reaction signal detection, and integrated the signal strength distribution for each accordingly.

This study found that the number of reports related to drug-associated dementia has steadily increased over time, with particularly high numbers since 2020 and peaking in 2021. This trend underscores the growing significance of drug-related dementia as a prominent drug safety concern. In addition, the majority of reports come from North America (63.20%), far exceeding those from Europe, Asia, and South America combined (27.93%), with nearly half originating from the United States. This regional discrepancy may reflect differences in disease epidemiology, drug utilization patterns, and medical practices among different regions. Notably, dementia-related AEs primarily occur in individuals aged 65 and above, with 46.72% of AE reports falling within this age group. Furthermore, the number of AE reports in females surpasses that in males by 12.21%. This not only indicates that drug-related dementia predominantly affects the elderly population, particularly females, but also indirectly reflects the positive correlation between the increasing number of dementia-related reports and population aging. Studies predict that by 2050, after age-standardization, there will be only a 0.1% statistical change in the prevalence of dementia in both males and females globally, suggesting a relative stability in dementia prevalence within the global age-sex ratio.^[12]^ The majority of AE reports are submitted by consumers (56.20%), followed by physicians (19.71%) and pharmacists (11.26%), indicating an enhanced consumer awareness of dementia. Overall, factors such as time, geography, and reporters may influence the reporting rate of dementia-related AEs. In addition, the clinical outcomes of most AE reports are severe (92.47%), with hospitalization accounting for 28.29% and death for 14.79%, highlighting the significant threat that dementia poses to patient health. Epidemiological data suggest that dementia is a significant contributor to mortality, emphasizing the importance of vigilance and preventive measures against these AEs in clinical practice.

Among the top 30 drugs related to dementia, psycholeptics, ISA, antithrombotic agents, and drugs for the treatment of bone diseases constitute the 4 categories with the highest number of reports. It should be emphasized that a higher number of reports in the FAERS database merely reflects the reporting distribution and may reflect greater drug utilization. It does not necessarily correspond to a higher risk in real-world clinical practice. With the annual increase in the prevalence of psychiatric disorders and the widespread use of antipsychotics (AP), a myriad of associated AEs has emerged.^[13,14]^ Pasina et al conducted a retrospective cross-sectional study, revealing that AP is extensively and frequently used in elderly patients.^[15]^ AP exhibits a strong affinity for dopamine D2 receptors and muscarinic M1 receptors, exerting inhibitory effects on both, which may adversely affect higher cognitive functions. This receptor affinity may be one reason for the heightened risk of dementia associated with AP.^[16]^ Studies have demonstrated that long-term use of any type of AP significantly increases the risk of dementia, notably raising its incidence among individuals under the age of 65.^[17,18]^ In addition, there exists a positive dose–response relationship between long-term oral AP and the risk of dementia, indicating that the cumulative use of AP is a crucial factor leading to pathological changes in the brain.^[17]^ Furthermore, first-generation AP and some second-generation AP can worsen cognitive function in elderly dementia patients, increasing the risks of death and cerebrovascular events.^[19,20]^ Moreover, Zheng et al found that anticholinergic drugs, including antiparkinson drugs and antidepressant drugs, increase the risk of dementia by 120%, with a dose-dependent relationship observed.^[21]^ Therefore, in an environment where most neurologic medications are used off-label, it is particularly important to thoroughly assess the risk-benefit ratio of drug use.

ISA is frequently used in the management of rheumatic immune disorders, especially rheumatoid arthritis and systemic lupus erythematosus.^[22]^ Research suggests that rheumatoid arthritis and systemic lupus erythematosus may elevate the risk of cognitive impairment through mechanisms such as chronic inflammation, atherosclerotic changes, and elevated antiphospholipid antibodies.^[23,24]^ In addition, there have been reports describing a case of a patient with ankylosing spondylitis induced by adalimumab who subsequently developed dementia.^[25]^ Consistent with our findings, conventional synthetic disease-modifying antirheumatic drugs (DMARDs) have been linked to a heightened risk of dementia.^[26]^ However, studies by Newby et al have found that long-term use of methotrexate gradually reduces the risk of dementia.^[27]^ Moreover, evidence regarding the dementia risk associated with biologic DMARDs and targeted synthetic DMARDs remains limited.^[28,29]^ Therefore, further investigation is warranted to explore the specific mechanisms underlying the comprehensive impact of rheumatic immune diseases and ISA on dementia risk. Healthcare professionals should develop personalized dosing regimens when prescribing ISA for patients with rheumatic immune disorders and remain vigilant regarding the potential risk of dementia.

## 5. Limitation

Although this study provides a comprehensive analysis of drug-associated dementia signals using a large-scale pharmacovigilance database, several limitations remain. The FAERS database is a spontaneous reporting system, and reports may be submitted by healthcare professionals, patients, and other individuals. Therefore, it is subject to several limitations, including underreporting, duplicate reporting, reporting bias, incomplete information, and data inaccuracies. In addition, report counts in FAERS may be influenced by multiple factors, such as the extent of drug use, disease prevalence, media or clinical attention, and differences in reporting practices. Consequently, a higher ranking in the number of reports does not necessarily indicate a higher true clinical risk, and the findings should be interpreted with caution. Moreover, because FAERS lacks denominator information, such as the total number of exposed individuals or the size of the treated population, this study could not calculate the true incidence of drug-related dementia or directly compare the risks of AEs across different drugs.

It should be emphasized that disproportionality analysis is primarily intended for pharmacovigilance signal detection. Therefore, reports in such databases only reflect statistical associations between drugs and dementia and cannot establish causal relationships. Further long-term studies are required for validation. In addition, clinical information in FAERS, including patients’ baseline diseases, comorbidities, and dementia subtypes, may be missing or incompletely documented. For example, some drug signals may be affected by confounding by indication. In particular, 8 antidementia medications, such as rivastigmine and donepezil, are used to treat Alzheimer’s disease and other dementia-related cognitive disorders. Therefore, the observed dementia-related reporting signals for these drugs may reflect the combined effects of drug exposure, the underlying disease, and disease progression. Importantly, the ROR/PRR magnitudes of such indication-confounded drugs should not be directly compared with those of drugs without dementia-related treatment indications as measures of relative clinical risk. Because FAERS lacks information on baseline cognitive status, disease severity, and other relevant clinical characteristics, this confounding cannot be adequately controlled, and the corresponding findings should be interpreted cautiously in the context of clinical practice and existing evidence.

Furthermore, the specific mechanisms underlying the association between various drugs and dementia-related AEs, particularly certain ISA, antithrombotic agents, and drugs for the treatment of bone diseases, remain unclear. Future prospective and multicenter clinical studies based on retrospective and cross-sectional studies are needed to elucidate the specific mechanisms in detail. Finally, elderly patients often present with multiple comorbidities, and polypharmacy is common in this population. Investigating the mechanisms of drug–drug interactions is crucial for determining the precise dementia risk associated with different medications, thus ensuring rational drug use and patient safety.

## 6. Conclusion

This study analyzed FAERS database records from the first quarter of 2004 to the first quarter of 2024 to identify potential drugs associated with disproportionate reporting of dementia-related AEs. Notably, drugs with the highest number of reports included apixaban, pimavanserin, and lenalidomide. This study provides valuable insights for identifying drugs with disproportionate dementia-related reporting signals in real-world scenarios and offers pharmacovigilance evidence to aid in the prevention and treatment of drug-related dementia, thereby enhancing the safety of drug therapy and optimizing clinical practice. However, our research has limitations, as AE signals in the FAERS database indicate statistical associations rather than direct causation. Therefore, while these signals can guide drug selection and usage in clinical settings, they should not replace the professional judgment of psychiatrists and clinical pharmacists. Future studies are needed to validate the direct causal relationship between specific drugs and dementia, providing more definitive guidance for clinical practice and drug regulation. Overall, the findings of this study should be regarded as hypothesis-generating pharmacovigilance evidence rather than confirmatory evidence.

## Author contributions

**Investigation:** Tiantian Tao, Bingyao Liu, Ruixiao Li.

**Methodology:** Tiantian Tao, Ke Wang.

**Formal analysis:** Bingyao Liu, Weiyi Chen.

**Conceptualization:** Chao Tao, Shiying Mao, Meiling Zhou, Xianbin Liu.

**Project administration:** Xianbin Liu.

**Supervision:** Xianbin Liu.

**Writing – original draft:** Tiantian Tao, Bingyao Liu.

**Writing – review & editing:** Chao Tao, Meiling Zhou.







## References

[1] Battini V Cirnigliaro G Giacovelli L. Psychiatric and non-psychiatric drugs causing false-positive amphetamines urine test in psychiatric patients: a pharmacovigilance analysis using FAERS. Expert Rev Clin Pharmacol. 2023;16:453–65. 10.1080/17512433.2023.2211261 37147189

[2] Khaleel MA Khan AH Ghadzi SMS Adnan AS Abdallah QM. A standardized dataset of a spontaneous adverse event reporting system. Healthcare (Basel). 2022;10:420. 10.3390/healthcare10030420 PMC8954498 35326898

[3] Song H Pei X Liu Z. Pharmacovigilance in China: evolution and future challenges. Br J Clin Pharmacol. 2023;89:510–22. 10.1111/bcp.15277 PMC10078654 35165914

[4] Ayhan Y Yoseph SA Miller BL. Management of psychiatric symptoms in dementia. Neurol Clin. 2023;41:123–39. 10.1016/j.ncl.2022.05.001 36400551

[5] Vuic B Milos T Tudor L. Pharmacogenomics of dementia: personalizing the treatment of cognitive and neuropsychiatric symptoms. Genes. 2023;14:2048. 10.3390/genes14112048 PMC10671071 38002991

[6] Jia L Du Y Chu L. Prevalence, risk factors, and management of dementia and mild cognitive impairment in adults aged 60 years or older in China: a cross-sectional study. Lancet Public Health. 2020;5:e661–71. 10.1016/S2468-2667(20)30185-7 33271079

[7] Zhao W Zhao L Chang X Lu X Tu Y. Elevated dementia risk, cognitive decline, and hippocampal atrophy in multisite chronic pain. Proc Natl Acad Sci U S A. 2023;120:e2215192120. 10.1073/pnas.2215192120 PMC9992778 36802440

[8] Alty J Farrow M Lawler K. Exercise and dementia prevention. Pract Neurol. 2020;20:234–40. 10.1136/practneurol-2019-002335 31964800

[9] Lynch C. World Alzheimer report 2019: attitudes to dementia, a global survey: public health: engaging people in ADRD research. Alzheimer’s Dement. 2020;16:e038255.

[10] Guzzon A Rebba V Paccagnella O Rigon M Boniolo G. The value of supportive care: a systematic review of cost-effectiveness of non-pharmacological interventions for dementia. PLoS One. 2023;18:e0285305. 10.1371/journal.pone.0285305 PMC10180718 37172047

[11] Wang C Song P Niu Y. The management of dementia worldwide: a review on policy practices, clinical guidelines, end-of-life care, and challenge along with aging population. Biosci Trends. 2022;16:119–29. 10.5582/bst.2022.01042 35466154

[12] Nichols E Steinmetz JD Vollset SE. Estimation of the global prevalence of dementia in 2019 and forecasted prevalence in 2050: an analysis for the global burden of disease study 2019. Lancet Public Health. 2022;7:E105–E25. 10.1016/S2468-2667(21)00249-8 PMC8810394 34998485

[13] Claassen JN Park JS. Examining the dispensing patterns of antipsychotics in Australia from 2006 to 2018 – a pharmacoepidemiology study. Res Soc Admin Pharm. 2021;17:1159–65. 10.1016/j.sapharm.2020.09.001 32928657

[14] Lee AK Lee SJ Dublin S. Disease latency and new-user versus prevalent-user cohort designs: implications for pharmacoepidemiology in dementia reply. J Am Geriatr Soc. 2024;72:956–7. 10.1111/jgs.18711 38147504

[15] Pasina L Novella A Cortesi L Nobili A Tettamanti M Ianes A. Drug prescriptions in nursing home residents: an Italian multicenter observational study. Eur J Clin Pharmacol. 2020;76:1011–9. 10.1007/s00228-020-02871-7 32314001

[16] Ma LY Ou YN Gao PY. Associations between antipsychotics exposure and dementia risk: a prospective cohort study of 415,100 participants. J Affect Disord. 2024;349:201–9. 10.1016/j.jad.2024.01.029 38199419

[17] Lowe XR Merchant M Whitmer RA. Antipsychotic medication risk of dementia and death: a propensity matched cohort study. Curr Drug Res Rev. 2022;14:139–47. 10.2174/2589977514666220301102717 35232344

[18] Mueller C John C Perera G Aarsland D Ballard C Stewart R. Antipsychotic use in dementia: the relationship between neuropsychiatric symptom profiles and adverse outcomes. Eur J Epidemiol. 2021;36:89–101. 10.1007/s10654-020-00643-2 PMC7847435 32415541

[19] Norgaard A Jensen-Dahm C Wimberley T. Effect of antipsychotics on mortality risk in patients with dementia with and without comorbidities. J Am Geriatr Soc. 2022;70:1169–79. 10.1111/jgs.17623 35029305

[20] Rogowska M Thornton M Creese B. Implications of adverse outcomes associated with antipsychotics in older patients with dementia: a 2011-2022 update. Drugs Aging. 2023;40:21–32. 10.1007/s40266-022-00992-5 PMC9747539 36513918

[21] Zheng YB Shi L Zhu XM. Anticholinergic drugs and the risk of dementia: a systematic review and meta-analysis. Neurosci Biobehav Rev. 2021;127:296–306. 10.1016/j.neubiorev.2021.04.031 33933505

[22] Cometi L Francesconi P Bellini B. Rheumatic diseases in migrant patients resident in Tuscany: epidemiological data analysis and single-center experience. Reumatismo. 2024;76:43–51. 10.4081/reumatismo.2024.1547 38523585

[23] Umoh IO dos Reis HJ de Oliveira ACP. Molecular mechanisms linking osteoarthritis and Alzheimer’s disease: shared pathways, mechanisms and breakthrough prospects. Int J Mol Sci. 2024;25:3044. 10.3390/ijms25053044 PMC10931612 38474288

[24] Wang YC Lin MS Huang APH Wu CC Kung WM. Association between systemic rheumatic diseases and dementia risk: a meta-analysis. Front Immunol. 2022;13:1054246. 10.3389/fimmu.2022.1054246 PMC9682025 36439141

[25] Hou C Azzi E Salmon A Osmont MN Perdriger A. Reversible anti-TNFα treatment induced dementia: a case report. Joint Bone Spine. 2021;88:105044. 10.1016/j.jbspin.2020.06.017 32622041

[26] Elahi N Ali A Idrees R. Does methotrexate use increase the risk of dementia in patients with rheumatoid arthritis? Int J Clin Pharmacol Ther. 2023;61:374–5. 10.5414/CP204433 37288837

[27] Newby D Prieto-Alhambra D Duarte-Salles T. Methotrexate and relative risk of dementia amongst patients with rheumatoid arthritis: a multi-national multi-database case-control study. Alzheimers Res Ther. 2020;12:38. 10.1186/s13195-020-00606-5 PMC7137292 32252806

[28] Desai RJ Varma VR Gerhard T. Comparative risk of Alzheimer disease and related dementia among medicare beneficiaries with rheumatoid arthritis treated with targeted disease-modifying antirheumatic agents. JAMA Netw Open. 2022;5:e226567. 10.1001/jamanetworkopen.2022.6567 PMC8994126 35394510

[29] Xie W Hou Y Xiao S Zhang X Zhang Z. Association between disease-modifying antirheumatic drugs for rheumatoid arthritis and risk of incident dementia: a systematic review with meta-analysis. RMD Open. 2024;10:e004016. 10.1136/rmdopen-2023-004016 PMC10900342 38413170

