# Supplementary material for: A FAERS pharmacovigilance analysis of dementia-related adverse event reports
Source: Medicine (Baltimore). 2026 Oct 16;105(42):e51134. doi: 10.1097/MD.0000000000051134 (PMC13681168; doi:10.1097/MD.0000000000051134)
Supplement: Supplementary file 1 [file medi-105-e51134-s002.docx]

**Supplemental Digital Content for**

**A FAERS Pharmacovigilance Analysis of Dementia-Related Adverse Event Reports**

**Table S1**. The classic two-by-two contingency table.

| Datebase | Number of reports for target AEs | Number of reports for other AEs | Total |
| --- | --- | --- | --- |
| Target drugs | a | b | a + b |
| Other drugs | c | d | c + d |
| Total | a + c | b + d | a + b + c + d |
